# Gastrointestinal schwannomas: a rare but important differential diagnosis of mesenchymal tumors of gastrointestinal tract

**DOI:** 10.1186/s12893-018-0379-2

**Published:** 2018-07-25

**Authors:** Alexandros Mekras, Veit Krenn, Aristotelis Perrakis, Roland S Croner, Vasileios Kalles, Cem Atamer, Robert Grützmann, Nikolaos Vassos

**Affiliations:** 1 Department of Surgery, University Hospital Erlangen, Krankenhausstrasse 12, 91054 Erlangen, Germany; 2 Department of Surgery, S. Elisabeth Hospital, Bernkastel/Wittlich, Germany; 3 MVZ-Zentrum für Histologie, Zytologie und Molekulare Diagnostik, Trier, Germany

**Keywords:** Schwannoma, Gastrointestinal, Mesenchymal tumor

## Abstract

**Background:**

Schwannomas of gastrointestinal tract are rare, mostly benign and notably different neoplasms from conventional schwannomas that arise in soft tissue or the central nervous system. These tumors are of clinical importance since they should always be considered in the differential diagnosis of submucosal lesions of gastrointestinal tract.

**Methods:**

Seven patients with a pathologically proven gastrointestinal schwannoma were identified in our series of mesenchymal tumors and reviewed retrospectively. Clinicopathological and immunohistochemical parameters along with the follow-up results were analysed.

**Results:**

The series included two males and five females, with a mean age 69 years (range, 39–81). Most patients were asymptomatic on presentation, except for two patients with abdominal pain. In the other cases (*n* = 5), the tumor was an incidental finding during other medical, imaging or surgical procedures. The tumors were located in the stomach (*n* = 4) and in the small intestine (*n* = 3) with an average size of 29 mm (range, 12–70). A preoperative diagnosis was achieved only in one case with a CT-guided core biopsy. Otherwise the clinical, intraoperative, endoscopic or radiological findings were unspecific. Patients with gastric tumor underwent either laparoscopic (*n* = 2) or open (*n* = 2) gastric wedge resection of the tumor; in the cases of intestinal tumor (*n* = 3) a segmentectomy was performed. Pathological examination revealed solid homogenous tumors, which were highly cellular and composed of spindle cells with positive staining for S100 protein, and confirmed the diagnosis of schwannoma. All tumors were negative for c-Kit, smooth muscle actin, desmin and DOG-1 and showed very low proliferation index. There were negative resection margins and no malignant variants were recognized. At an average follow-up of 60 months (range, 24–185) all patients were free of disease with no signs of recurrence or metastases and acceptable gastrointestinal function.

**Conclusions:**

Schwannomas are rare, slow-growing and mostly asymptomatic gastrointestinal mesenchymal tumors. They are difficult to be diagnosed preoperatively as endoscopic and radiological findings are nonspecific but histological and immunohistochemical features are of paramount importance to differentiate between benign and malignant schwannomas, or other spindle cell sarcomas. The treatment of choice is complete surgical excision without a conclusive preoperative diagnosis, and the long-term outcome is excellent as these lesions are mostly benign.

## Background

Schwannomas are slow-growing, homogeneous, mostly benign tumors arising from the schwann cells of the nerve sheath [1, 2]. They are most commonly found in the cranial vault including the myelin-forming cells of the 8th cranial nerve [1, 3]. They can rarely occur in the gastrointestinal (GI) tract, representing about 2–6% of all mesenchymal tumors [4]. The most common site (60–70% of all GI cases) is the stomach, followed by the colon and rectum (3%) [5–10]. Small-intestinal and esophageal schwannomas have been very rarely reported [7].

Schwannomas of the GI tract, firstly presented by Daimaru et al. in 1988 [11], are usually benign and notably different neoplasms from conventional schwannomas that arise in the soft tissue or the central nervous system [7]. They are classified under a heterogeneous group of mesenchymal or neuroectodermal neoplasms which arise from the wall of the gastrointestinal tract and include schwannomas, gastrointestinal stromal tumors (GIST), leiomyomas, leiomyosarcomas, neurofibromas, lipomas, ganglioneuromas, paragangliomas, granular cell tumors, and glomus tumors [7]. The GI schwannomas are most frequently asymptomatic presenting as submucosal lesions, usually detected incidentally during a laparoscopy / laparotomy, esophagogastroduodenoscopy (EGD), endoscopic ultrasonography (EUS) or at imaging [6, 7]. A preoperative diagnosis of schwannomas cannot always be carried out with high accuracy and the diagnosis is established by the pathological examination of the surgical specimen [5].

The purpose of our study was the description of clinical, histopathological and immunohistochemical features of GI schwannomas, providing long-term follow-up and confirming the benign nature of these tumors.

## Methods

Seven patients with histologically identified GI schwannoma during a 10 year period, were involved in this retrospective study. Clinical, histopathological, immunohistochemical and surgical data were reviewed. A complete surgical excision of the tumors was performed in all patients. Specimens were obtained from all patients. The diagnosis of GI schwannomas was based on histopathological analysis accompanied by immunohistochemical staining performed for S-100 protein, glial fibrillary acidic protein (GFAP), c-kit (CD117), CD34, discovered on GIST-1 (DOG-1), smooth muscle actin (SMA) and desmin. The patients were regularly followed up on an outpatient basis. Follow-up included physical examination, blood counts, serum chemistries, endoscopy and computed tomography (CT)-scan. Median follow-up was 60 months (range, 24–185).

## Results

### Clinical features and preoperative evaluation

**Fig. 1:**
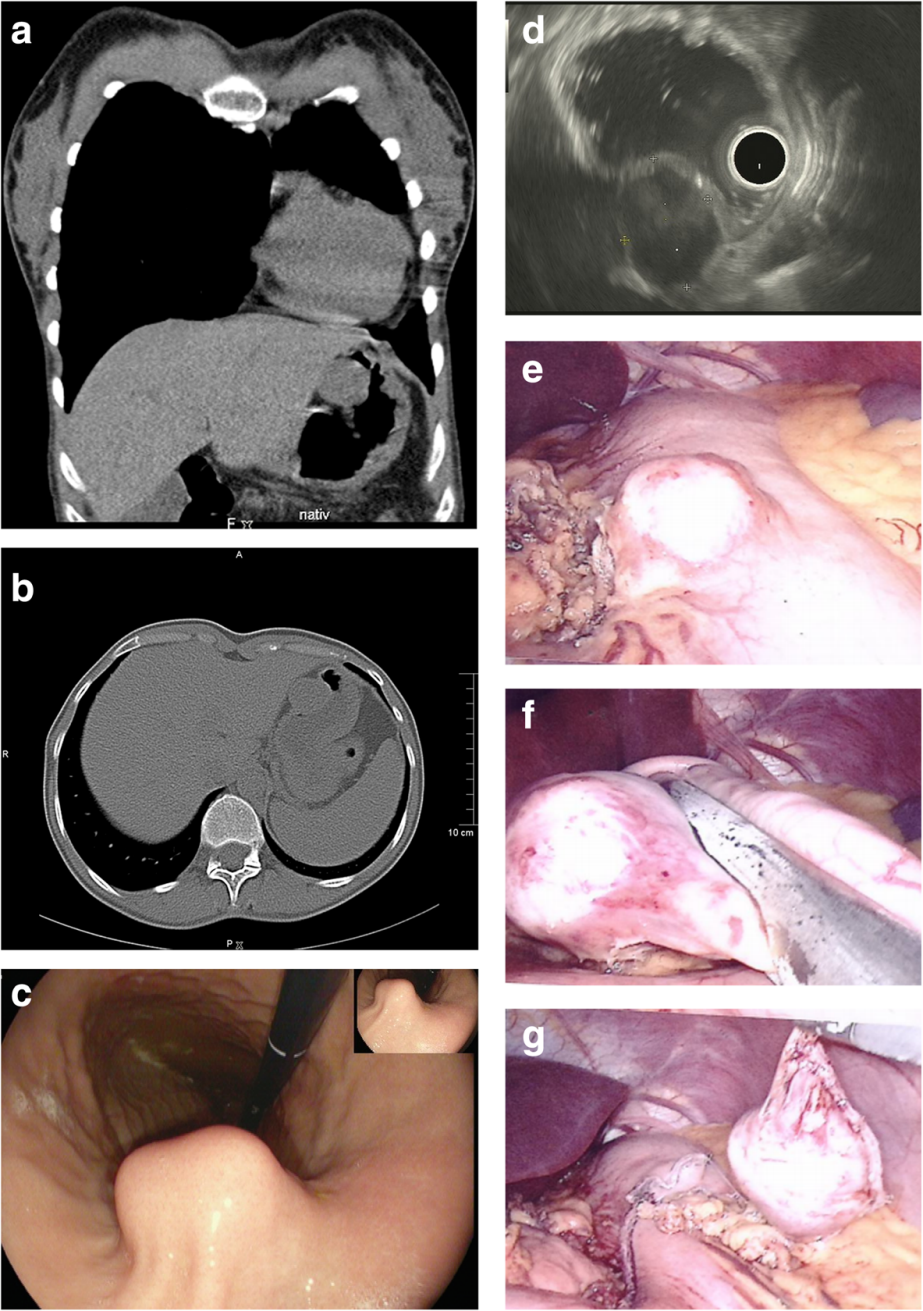
**a, b.** Incidental contrast-enhanced computed tomography (CT) finding shows a round, exophytic, well-defined, homogeneous mass (30 mm) with contrast enhancement, arising from the lesser curvature of the gastric body. **c** GI endoscopy submucosal mass (26 mm × 22 mm) with smooth overlying normal mucosa in the lesser curvature of the gastric body. **d** EUS picture showing a hypoechoic lesion located within the muscularis propria. **e** Laparoscopic intraoperative picture of an exophytic mass along the lesser curvature of the body of the stomach. **f, g** Laparoscopic tumor wedge resection using mechanical laparoscopic sutures

The demographic and clinicopathologic data of our patients are summarized in Table 1. Five women and two men, with a median age of 69 years (range, 39–81) were diagnosed with a schwannoma of the GI tract. The tumor was localized to the stomach in four patients (*n* = 4) and in the small intestine in three patients (*n* = 3), with a median tumor size of 29 mm (range, 12–70). Out of the four gastric schwannomas, two were localized in the gastric body and two in the antrum. Symptoms were reported in only one patient with gastric schwannoma who complained of persistent epigastric pain and nausea. In six cases the tumor was an incidental finding during imaging, endoscopic or surgical procedures for other reason. Particularly the tumors were incidentally discovered during surgery for colon carcinoma (case Nr. 1) and diverticulitis of sigmoid (case Nr. 3, 4), during an abdominal ultrasound as preventative measure (case Nr. 2) and during a upper GI gastroscopy performed as preventative measure as well (case Nr. 5). Furthermore, in the seventh patient, the tumor was an incidental finding on a thoracic CT scan performed for evaluation of hemoptysis. CT scan revealed a round homogeneous mass with contrast enhancement and a predominantly exophytic pattern arising from the lesser curvature of stomach (Fig. 1a, b)*.* None of the patients had a history of neurofibromatosis.

**Table 1:** Demographical and clinicopathological data of our cases with GI schwannomas

Case	Age/Sex	Presentation	Location	Operation	Size/ mm	Follow up
1	73/F	Incidental finding during operation	Gastric body	Gastric wedge resection	25	DF/ 185 m
2	70/F	Incidental finding during routine US	Duodenum	Local tumor resection	22	DF/ 90 m
3	72/F	Incidental fining during operation	Ileum	Small bowel segmentectomy	70	DF/ 42 m
4	81/M	Incidental finding during operation	Jejunum	Small bowel segmentectomy	12	DF/ 21 m
5	72/M	Incidental finding during upper GI endoscopy	Antrum	Gastric wedge resection	16	DF /21 m
6	77/F	Epigastric pain/ upper GI endoscopy	Antrum (greater curvature)	Laparoscopic gastric wedge resection	15	DF/ 20 m
**7**	39/F	Incidental finding during thoracic CT	Gastric body (lesser curvature)	Laparoscopic gastric wedge resection	25	DF/ 24 m

*M* Male, *F* Female, *DF* Disease Free, *m* months, *US* Ultrasound, GI Gastrointestinal

An upper gastrointestinal endoscopy was carried out in 3/4 of the cases with gastric schwannoma, which demonstrated submucosal gastric tumors with smooth overlying mucosa and without any ulceration (Fig. 1c), ranging from 12.5 mm to 25 mm. For further diagnostic evaluation, in two of the gastric cases an EUS was performed, in which a heterogeneous hypoechoic submucosal tumor arising from the muscularis propria of the stomach was demonstrated (Fig. 1d). Biopsy was attempted but it was nondiagnostic, suggesting mostly a stromal tumor with nonspecific spindle cells. A preoperative diagnosis of schwannoma was obtained only in one case (small intestine schwannoma) with a CT-guided needle biopsy, which was performed because of the adherence of tumor with the pancreas and its unclear entity (case Nr. 3).

### Surgical, histopathological and immunochemical features

All patients underwent a tumor resection with negative resection margins (R0). Patients with gastric schwannomas (*n* = 4) underwent a gastric wedge resection by using either an open (*n* = 2) or a laparoscopic approach (*n* = 2) (Fig. 1e-g). In both laparoscopic cases the exophytic mass was located along the lesser and the greater curvature of the stomach and was easily detected and clearly identified by the laparoscope. Patients with intestinal schwannomas (*n* = 3) received a small bowel segmentectomy (segmental resection). The median tumor size was 26.5 mm (range, 12–70 mm). Neither morbidity nor mortality was observed in our series.

**Fig. 2:**
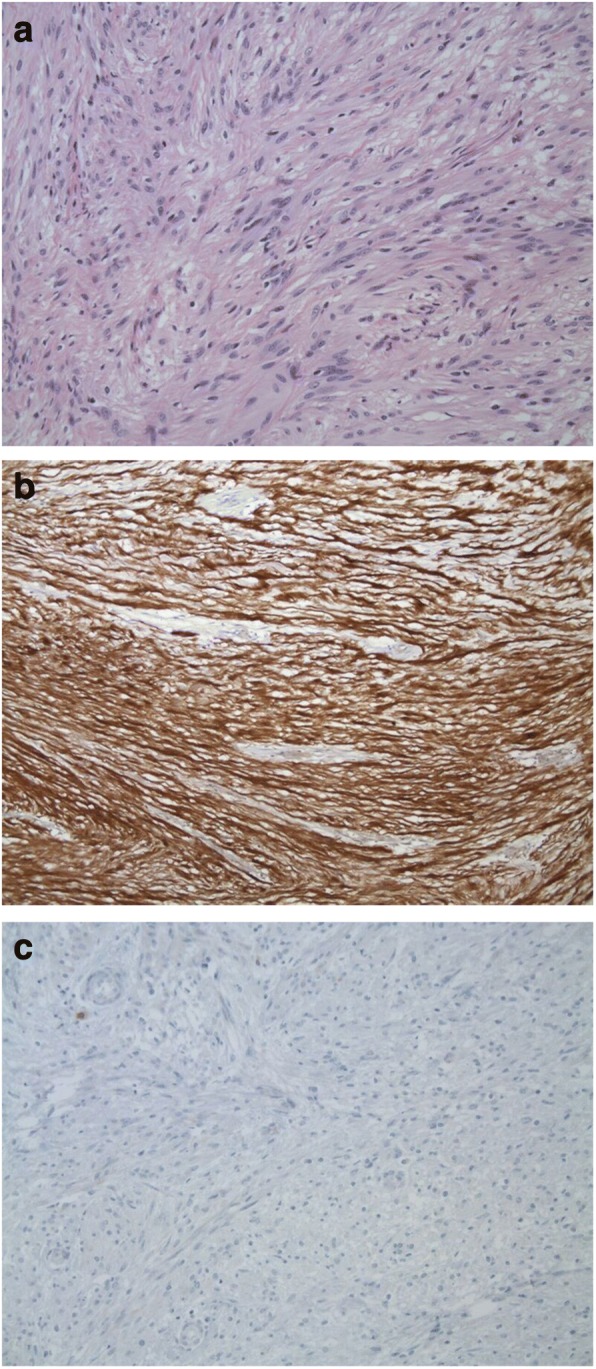
Representative illustrations from GI schwannoma cases. **a** The tumor cells are spindle shaped with elongated nuclei and form loose interlacing fascicles or whorls (HE, original magnification × 250). **b** Intense expression of S100 immunostain in tumor cells (DAB magnification × 250). c CD117 absence of staining in tumor population (DAB magnification × 250)

The histopathologic diagnosis of schwannoma was confirmed in all patients. Pathological examination demonstrated the GI schwannomas to be solid homogenous tumors, which were highly cellular and composed of spindle cells. No areas of cystic change of gross necrosis were reported in any of the cases. Mitotic activity was very low (< 5/50 high power fields). All specimens demonstrated strong positive immunostaining for S-100 and GFAP proteins, whereas the tumor cells were not immunoreactive to DOG1, c-Kit (CD117), CD34, SMA and desmin (Fig. 2).

#### Follow-up

Median follow-up was 60 months (range, 24–185). During the follow-up period no recurrence was detected. Furthermore no excessive body weight loss and no gastrointestinal symptoms such as dyspepsia and bloating were observed.

## Discussion

Gastrointestinal schwannomas are rare mesenchymal tumors [5–7], firstly reported by Daimaru, who identified a schwannoma as a primary GI tumor entity based on the positive S-100 immunostaining [11]. They represent ca. 2 to 6% of all mesenchymal tumours of the GI tract [4, 12, 13]. They are of clinical importance since GI schwannomas are notably different neoplasms from conventional soft-tissue and central nervous system schwannomas, some of which may be associated with neurofibromatosis 2.

In terms of pathologic evaluation, GI schwannomas are classified as non-epithelial tumours, and represent a separate, homogenous entity, distinct from leiomyomas, leiomyosarcomas, gastrointestinal autonomic nerve tumors (GANTs) and GISTs [12, 14, 15]. They can be identified in every part of the GI tract but they occur predominantly in stomach. The second most common site seems is colon, whereas schwannomas located to small intestine or esophagus are rare [5–9, 16, 17].

Our series shows a remarkable regularity regarding to the characteristics of GI-schwannomas. They occur more frequently in the sixth decade of life and there is a predominance of females [11, 12, 15, 17–21]. Our results showed that all of our patients except one woman (39 years-old), were over 70 years of age at the time of diagnosis. These tumors do not usually cause any symptoms and can be found out incidentally, as presented in our series. However, when symptomatic, they represent with unspecific clinical symptoms, and particularly Bruneton et al. reported that most common symptoms of these patients are bleeding and abdominal pain [22].

A preoperative diagnosis of GI schwannomas is generally difficult and challenging, if not impossible, since the tumor have no specific clinical symptoms and no diagnostic modality can show any pathognomonic features unique to this tumor [22]. On CT examination, these tumors mostly represent as exophytic masses displaying homogeneous enhancement in most cases whereas cystic change, cavity formation, necrosis or calcification are uncommon [7, 16, 24]. Our CT findings were comparable to previous reports in terms of enhancement patterns and growth patterns. On endoscopy, gastric schwannomas appear grossly as elevated submucosal lesions, and a central ulcer can be seen in 25–50% due to ischemic changes in the covering mucosa [16, 25]. It should be noted that there is an important false-negative rate of endoscopic biopsy diagnosis because of normal mucosa overlying a submucosal lesion [22]. Therefore, endoscopic biopsy is supposed to be not as effective as expected in the diagnosis of gastric schwannomas, since the results mostly demonstrate nonspecific spindle cells. EUS-guided fine needle aspiration (FNA) biopsies of submucosal lesions in the upper GI tract can be helpful in preoperative diagnosis of spindle cell tumors but are sometimes not representative of deeper submucosal tissue [25–29]. Two patients with gastric schwannomas underwent an EUS-guided FNA without representative findings. However, in one case (case Nr. 3) a CT-guided biopsy of a 2.2 cm mass of duodenum was performed and detected a GI schwannoma. Generally, the CT and endoscopic findings in our patients were similar to those from previous studies as far as the location and mucosal change.

GI-schwannomas are supposed to arise from the myenteric plexus of the GI wall because of their immunophenotypic similarities; both schwannomas and myenteric plexus cells express S-100 protein and GFAP [7, 11]. Pathologically, GI schwannomas are considered to be notably different tumors from conventional schwannomas, which arise from central nervous system and soft tissues [7, 21]. Macroscopically, these tumors are round or fusiform and their cut surface reveals smooth, glistening, grey-white appearance. Microscopically, unlike conventional schwannomas, GI schwannomas are not always encapsulated, but mostly well circumscribed. Cystic areas of hemorrhage and calcification may be present. These tumors are composed of uniformly spindled schwann cells arranged in a interlacing fascicles, often with germical centers [4, 21].

The pathologic findings of the GI schwannomas in the present analysis were consistent with the previously described findings. On immunohistochemistry, vimentin, GFAP and S100 protein are diffusely and strongly expressed by the cells of GI schwannomas [21]; the S100 immunostaining pattern is both in a nuclear and cytoplasmic distribution [4]. Expression of CD34 is rare, whereas there is lack of CD117, SMA and desmin positivity [30]. This immunohistochemical pattern is very important because it can differentiate GI schwannomas from other GI tract mesenchymal neoplasms.

The main differential diagnosis for an exophytic lesion arising in the wall of the GI tract is a GIST, as it is the most common mesenchymal tumor located in GI tract [7, 31, 32]. Voltaggio et al. estimated that the ratio of gastric GIST to gastric schwannoma is approximately 45 to 1 [33]. Some other smaller studies have shown lower gastric GIST frequencies (8-14 to 1) in relation to schwannoma [11, 19]. In these cases, immunohistochemistry is extremely helpful. Another tumor entity included in the differential diagnostic of GI schwannomas is the primary and secondary lymphomas because of their similar CT appearance to schwannomas. Both of them arise from the GI wall and tend to appear as a homogeneous mass in CT. However, the presence of adenopathy in most of lymphomas, in contrast to GI schwannomas, is an important distinguishing feature [7]. Other entities included in the differential diagnosis are GI variant of clear cell sarcoma, metastatic malignant melanoma and GI adenocarcinoma [7, 34].

Schwannomas of the GI tract are generally benign since previous follow-up studies have not identified any malignant variants [4, 9, 11, 19–21]. Therefore, pathologic parameters such as tumor size and mitotic rate seem not to have any prognostic significance. The complete surgical resection seems to be the treatment of choice because of the preoperative diagnostic uncertainty and the excellent long-term outcome, as these tumors are uniformly benign [23, 35]. Tumor recurrence is generally associated with an incomplete surgical margin. The outcome after surgical resection in our study was excellent with no malignant variant and no recurrences or metastases. However, a very few cases of gastric malignant schwannomas have been reported in the literature [25, 36, 37]. Malignant GI schwannomas are extremely rare and they cannot be distinguished from benign schwannomas only on the basis of the histopathological examination of the resected specimens. Furthermore, cases of “malignant schwannomas” also called malignant peripheral nerve sheath tumors have been reported [38]. These malignant tumors with neural differentiation are considered to be distinct tumors from GI schwannomas and are called gastrointestinal autonomic nerve tumors (GANTs) [38]. The usefulness of molecular therapy for gastric malignant schwannomas is not clearly established because only a few cases have been reported. Further molecular therapy studies will be useful in order to determine its role in the treatment of GI malignant schwannoma.

## Conclusions

Schwannomas are rare, slow-growing and mostly asymptomatic gastrointestinal mesenchymal tumors, notably different from conventional soft-tissue and central nervous system schwannomas. They present a challenge in GI tract surgical oncology due to difficulty in the preoperative diagnosis, as the clinical, endoscopic and imaging findings are nonspecific. The definitive diagnosis can only be established through the histopathological and immunohistochemical examination, which is confirmatory. Complete surgical excision is the gold standard in the treatment of schwannomas and the prognosis of patients is excellent since these lesions are mostly benign.

## Abbreviations

CT: Computed tomography
DOG-1: Discovered on GIST-1
EGD: Esophagogastroduodenoscopy
EUS: Endoscopic ultrasonography
FNA: Fine needle aspiration
GANT: Gastrointestinal autonomic nerve tumors
GFAP: Glial fibrillary acidic protein
GI: Gastrointestinal
GIST: Gastrointestinal stromal tumor
SMA: Smooth muscle actin
